# Transcriptional and Molecular Pathways Activated in Mesenteric Adipose Tissue and Intestinal Mucosa of Crohn's Disease Patients

**DOI:** 10.1155/2017/7646859

**Published:** 2017-04-09

**Authors:** Andressa Coope, Lívia Bitencourt Pascoal, Francesca Aparecida Ramos da Silva, José Diego Botezelli, Maria de Lourdes Setsuko Ayrizono, Marciane Milanski, Michel Gardere Camargo, Núria Planell, Mariana Portovedo, Cilene Bicca Dias, João José Fagundes, Raquel Franco Leal

**Affiliations:** ^1^IBD Research Laboratory, Surgery Department, University of Campinas (UNICAMP), Medical School, Campinas, SP, Brazil; ^2^Laboratory of Metabolic Disorders, Faculty of Applied Sciences, University of Campinas (UNICAMP), Campinas, SP, Brazil; ^3^Department of Gastroenterology and Bioinformatics Platform, CIBER-EHD, Barcelona, Spain

## Abstract

Crohn's disease (CD) is a chronic inflammatory disorder, characterized by cytokine imbalance and transcription signaling pathways activation. In addition, the increase of mesenteric adipose tissue (MAT) near the affected intestinal area is a hallmark of CD. Therefore, we evaluated the transcription signaling pathways and cytokines expression in intestinal mucosa and MAT of active CD patients. Ten patients with ileocecal CD and eight with noninflammatory diseases were studied. The biopsies of intestinal mucosa and MAT were snap-frozen and protein expression was determined by immunoblotting. RNA levels were measured by qPCR. The pIkB/IkB ratio and TNF*α* level were significantly higher in intestinal mucosa of CD when compared to controls. However, STAT1 expression was similar between intestinal mucosa of CD and controls. Considering the MAT, the pIkB/IkB ratio was significantly lower and the anti-inflammatory cytokine IL10 was significantly higher in CD when compared to controls. Finally, the protein content of pSTAT1 was higher in MAT of CD compared to controls. These findings reinforce the predominance of the proinflammatory NF-kB pathway in CD intestinal mucosa. For the first time, we showed the activation of STAT1 pathway in MAT of CD patients, which may help to understand the physiopathology of this immune mediated disease.

## 1. Introduction

Crohn's disease (CD) is characterized by mucosal immune cell activation and cytokine imbalance. The tumor necrosis factor alfa (TNF*α*) is one of the most important proinflammatory cytokines involved in this process, and its transcription depends on the activation of the transcription factor known as nuclear factor kB (NF-kB) [1]. Besides, some members of the signal transducer and activator of transcription (STAT) family have been involved in this process. STAT1 is upregulated in response to interferons and when activated (pSTAT1) binds to the promoter region of interferon-stimulated genes. All the transcription factors translocate into the nucleus and interact with conserved regulatory DNA sequences resulting in the transcription of genes like chemokines, cytokines, receptors, signaling regulatory genes, among others. Those molecules have been studied in several gastrointestinal disorders. However, the most common data about them comes from analysis of intestinal tissue, rarer than from the mesenteric adipose tissue (MAT) nearby the affected intestinal area [2]. The increase of the MAT is a common feature of the disease and may involve the small and large bowels; thus this tissue may represent a relevant role in the pathogenesis of CD [3, 4]. Histological analyses reveal abnormalities in the adipose tissue, including infiltration of macrophages and fibrosis [5]. Adipose tissue is able to release hormones which can lead to pro- or anti-inflammatory pathways, such as leptin that has been shown to increase the secretion of TNF*α* and IL6, as well as activate NF-kB [6, 7] and such as adiponectin and anti-inflammatory factors which attenuate the proinflammatory response.

TNF*α* has several functions, which are generally counterbalanced by anti-inflammatory pathways, such as IL10. There is an exacerbation of TNF*α* expression along with other inflammatory mediators in the intestinal* lamina propria* of the inflammatory bowel diseases (IBD). The intraluminal stimuli lead to the increased expression of interleukins such as IL1*β*, IL6, and IL8, which stimulate the proliferation and activation of lymphocytes, by a mechanism not completely understood [8].

Studies of immunological differences between CD and ulcerative colitis (UC), the two major IBD, have shown that the activation of NF-kB is more common in CD than in UC; conversely, expression and activation of STAT1 are predominantly high in UC. Indeed, STAT1 is activated by an intracytoplasmic pathway activated by the transmembrane receptor of IFN*γ* [9, 10] and can be blocked by SOCS3, which play an important role in the regulation of this pathway [11, 12].

This study aimed to investigate if the intestinal mucosa and MAT of CD patients activate different molecular and transcriptional pathways, which could lead to different tissue-specific drugs response in patients and, also, to establishing potential pharmacologic targets to treat CD. Thus, we evaluated molecules involved in the activation of NF-kB and STAT1 pathways and consequent cytokine expression in intestinal mucosa and MAT of active CD patients. Finally, the correlation between intestinal mucosa and MAT of CD patients may help towards a better understanding of the pathophysiology of this chronic inflammatory disorder.

## 2. Materials and Methods

### 2.1. Chemicals and Reagents

All the reagents for SDS-polyacrylamide gel electrophoresis and immunoblotting were from Bio-Rad Laboratories (Richmond, CA, USA). HEPES, phenylmethylsulfonyl fluoride, aprotinin, dithiothreitol, Triton X-100, Tween 20, glycerol, and BSA (fraction V) were purchased from Sigma Chemical Co. (St. Louis, MO, USA). Nitrocellulose paper (BA85, 0.2 *μ*m) and the reagents for chemoluminescence protein labeling in immunoblots were purchased from Amersham (Aylesbury, UK). The antibodies against IL10 (sc1783), I*κ*B*α* (sc1643), pIkK (sc7977), pSTAT1 (sc7988), TNF*α* (sc8301), and SOCS3 (sc7009) were from Santa Cruz Biotechnology (Santa Cruz, CA, USA). IL6 (ab 6672), IL17 (ab 79056), and IL23 (ab45420) antibodies were from Abcam (Cambridge, MA). The antibody against IL1*β* (503502) was obtained from Biolegend (San Diego, CA). The protein molecular weight was assessed by the PageRulerTM from Fermentas (Glenburnie, MD). Reagents for real-time PCR analysis were from Invitrogen (Carlsbad, CA, USA) and Applied Biosystems (Foster City, CA, USA). Taqman Primers for* TNFα* (Mm00443258_m1),* IL1β* (Mm00434228_m1),* IL6* (Mm00446190_m1),* IL10* (Mm01288386_m1), and glyceraldehyde-3-phosphate dehydrogenase (*GAPDH*) (#4352339E) were obtained from Applied Biosystems and Integrated DNA Technologies (IDT).

### 2.2. Experimental Protocols

Biopsies from intestinal mucosa (ICD Group) and from mesenteric adipose tissue (ACD Group) were taken from 10 patients with ileocecal CD who underwent surgical resection. The presence of disease activity was assessed by colonoscopy one day before surgery and all patients had Crohn's disease activity index (CDAI) [13] more than 250 points. Patients with CD in other locations were excluded. The control group of ileal tissue was composed of eight patients who underwent ileocolonoscopy and the examination was normal (intestinal mucosa tissue control group–IC Group). The control group of MAT was composed of eight patients who underwent rectosigmoidectomy for noninflammatory disease with normal distal ileum (ileum mesenteric adipose tissue control group–AC Group). The study was performed in accordance with the Declaration of Helsinki and was approved by the Ethical Committee of the University of Campinas. All samples were taken after informed consent from the patients. The study was carried out at the University of Campinas, IBD Research Laboratory of the Colorectal Surgery Unit, Surgery Department, and at the Laboratory of Cell Signaling of the Department of Internal Medicine.

### 2.3. Western Blot

Biopsies were snap-frozen in liquid nitrogen and stored at −80°C until use. For total protein extract preparation, the fragments were homogenized in solubilizing buffer at 4°C [1% Triton X-100, 100 mM Tris-HCl (pH 7.4), 100 mM sodium pyrophosphate, 100 mM sodium fluoride, 10 mM EDTA, 10 mM sodium orthovanadate, 2.0 mM phenylmethylsulfonyl fluoride (PMSF), and 0.1 mg aprotinin/ml] with a Polytron PTA 20S generator (model PT 10/35; Brinkmann Instruments, Westbury, NY) operated at maximum speed for 30 sec. Insoluble material was removed by centrifugation (12000 rpm at 4°C for 40 min). The protein concentration of the supernatants was determined by BCA method (Pierce™ BCA Protein Assay Kit. Catalog number 23225). Aliquots of the supernatants containing 50 *μ*g total proteins were separated by SDS-PAGE, transferred to nitrocellulose membranes and blotted with indicated antibodies as described in the results. Specific bands were labeled by a chemiluminescence reaction (SuperSignal West Pico Chemiluminescent Substrate from Pierce Biotechnology, Inc. Rockford, IL) and quantified by optical densitometry (Un-Scan-It Program). We have applied Ponceau staining to check equal loading of gels and membrane transfer (see Supplementary Figure S1 in Supplementary Material available online at https://doi.org/10.1155/2017/7646859) [14, 15].

### 2.4. RNA Extraction and Quantitative Real-Time PCR (qPCR)

Total RNA was extracted using a commercially available acid-phenol reagent Trizol (Invitrogen Corp.) according to the manufacturer's instructions. The RNA concentration, purity, and integrity were confirmed spectrophotometrically using a Nanodrop (ND-1000; Nanodrop Technologies, Wilmington, DE). RNA was treated with RNase-free Dnase (RQ1 RNase-free Dnase, Promega) and then reverse transcribed using oligo (dT) primers and reverse transcriptase (RevertAid™ Kit, Fermentas). The reaction mixture (20 *μ*l) was incubated at 42°C for 60 min and then 10 min at 70°C and cooled on ice. qPCR was performed on resulting cDNA, using the manufacturer's protocol. Amplification was performed in a 10 *μ*L final volume containing 40–50 ng of reverse-transcribed RNA according to the manufacturer's recommendations using the TaqMan PCR master mix.

Real-time PCR amplification consisted of an initial denaturation step (50°C for 2 min and 95°C for 10 min), 40 cycles of denaturation (95°C for 15 s), annealing (53°C for 20 s), and extension (72°C for 20 s), followed by a final incubation at 60°C for 1 minute. All measurements were normalized by the expression of* GAPDH* gene, considered as a stable housekeeping gene. Gene expression was determined using the delta-delta Ct method [16].

Real-time PCR analysis of gene expression was carried out in an ABI Prism 7500 sequence detection system (Applied Biosystems). The optimal concentration of complementary DNA (cDNA) and primers, as well as the maximum efficiency of amplification, were obtained through five-point, twofold dilution curve analysis for each gene. Amplification was performed in a 20 *μ*L final volume containing 40–50 ng of cDNA according to the manufacturer's recommendations using the TaqMan PCR master mix. Real-time data were analyzed using the Sequence Detector System  1.7 (Applied Biosystems). Results were expressed as relative transcript amount as previously optimized [16].

### 2.5. Histological Analysis: Hematoxylin and Eosin (H&E) Staining

Biopsies from the intestinal mucosa and from MAT near the affected intestinal area were embedded in paraffin blocks for histological analysis. Sections of 5 *μ*m were cut and stained with H&E dye. Photomicrographs were taken using a Zeiss Axiophot microscope and Cannon Power Shot G5 digital camera system (Cannon Inc., Tokyo). Fields of higher magnification (20x) were scanned and random fields were analyzed.

### 2.6. Statistical Analysis

All results are reported as means ± SEM. Data were analyzed by the Mann–Whitney Test, comparing the MAT of the CD group and its respective adipose control group and comparing, separately, the intestinal tissue of the CD group and its respective intestinal control group. The level of significance was set at *p* < 0.05.

## 3. Results

### 3.1. Morphometric Analysis of the Intestinal and MAT of CD Patients

**Figure 1:**
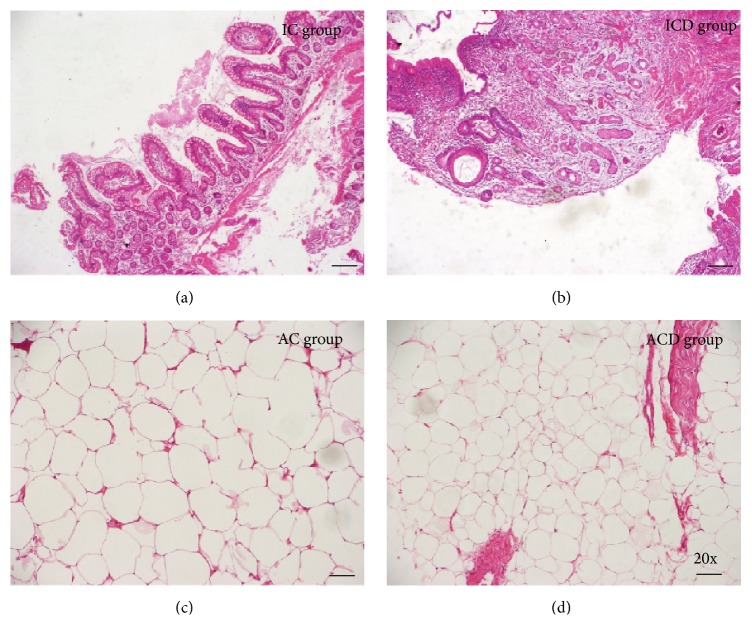
Haematoxylin and Eosin (H&E) staining of intestinal mucosa and mesenteric adipose tissues segments of a representative Crohn's disease patient. (a) Intestinal mucosa of a normal control subject (IC Group). (b) Intestinal mucosa of CD patient (ICD Group), showing crypt distortion, ulcers, and inflammatory infiltrate. (c) Mesenteric adipose tissue of a normal control subject (AC Group). (d) Mesenteric adipose tissue of CD patient (ACD Group) revealing characteristic morphometric features, with reduced adipocyte size. Nuclear counterstaining: Mayer's haematoxylin. Original magnification ×20.

H&E staining of CD intestinal mucosa showed thickening of the bowel wall associated with inflammation and deep linear ulcerations (Figure 1(b)) when compared to control (Figure 1(a)). In addition, we demonstrated that the area and the perimeter of the MAT were lower (Figure 1(d)) in CD patients when compared to the control group (Figure 1(c)).

### 3.2. Evaluation of the Inflammatory Markers in Intestinal Mucosa of CD Patients

**Figure 2:**
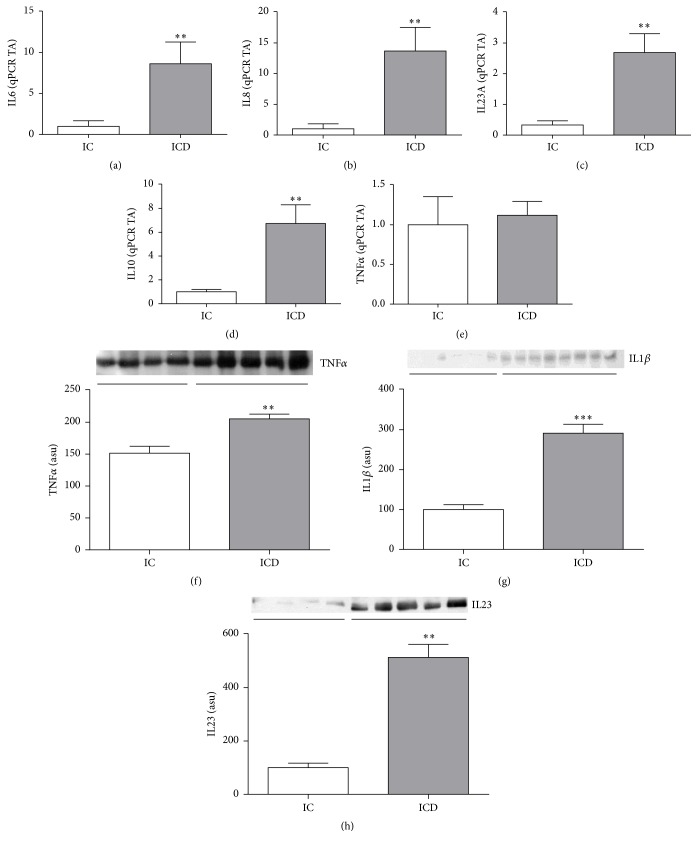
Evaluation of cytokines gene expressions in the intestinal mucosa of Crohn's disease patients. Inflammatory signaling proteins reveal a proinflammatory pattern in the intestinal mucosa of Crohn's disease patients. mRNA levels (qRT-PCR) of* IL6* (a),* IL8* (b),* IL23A* (c),* IL10* (d), and* TNFα* (e), in intestinal mucosa of CD patients (ICD Group) compared to controls (IC Group). Western blot analysis of TNF*α* (f), IL1*β* (g), and IL23 (h) in intestinal mucosa of CD patients (ICD Group) compared to controls (IC Group). Each band represents one patient. For ICD, *n* = 10; for IC, *n* = 8,  ^*∗*^*p* < 0.05,  ^*∗∗*^*p* < 0.01, and ^*∗∗∗*^*p* < 0.001 versus control. TA: transcription amount. ASU: arbitrary scanning unit.

Patients with CD showed increased mRNA levels of inflammatory markers such as* IL6, IL8, IL23,* and* IL10* (*p* < 0.05; Figures 2(a), 2(b), 2(c), and 2(d), resp.) in the intestinal mucosa when compared to control. However, no difference in* TNFα* gene expression was detected between ICD and IC groups (Figure 2(e)). On the other hand, the protein level of TNF*α*, IL1*β*, and IL23 (*p* < 0.05; Figures 2(f), 2(g), and 2(h), resp.) was significantly higher in the ICD Group when compared to control.

### 3.3. Evaluation of the Inflammatory Markers in MAT of CD Patients

**Figure 3:**
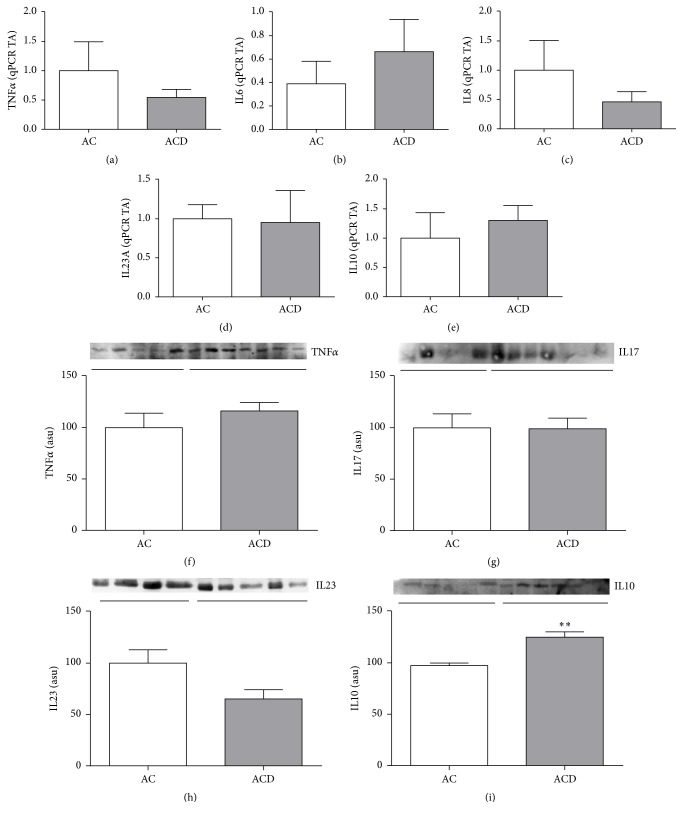
Evaluation of cytokines gene expressions in the mesenteric adipose tissue of Crohn's disease patients. Inflammatory signaling pattern in the mesenteric adipose tissue of Crohn's disease patients. mRNA levels (qRT-PCR) of* TNFα* (a),* IL6* (b),* IL8* (c),* IL23A* (d), and* IL10* (e) in the mesenteric adipose tissue (MAT) of CD patients (ACD Group) compared to controls (AC Group). Western blot analysis of TNF*α* (f), IL17 (g), IL23 (h), and IL10 (i) in the mesenteric adipose tissue (MAT) of CD patients (ACD Group) compared to controls (AC Group). Each band represents one patient. For ACD, *n* = 10; for AC, *n* = 8,  ^*∗*^*p* < 0.05,  ^*∗∗*^*p* < 0.01, and ^*∗∗∗*^*p* < 0.001 versus control. TA: transcription amount. ASU: arbitrary scanning unit.

*TNFα, IL6, IL8, IL23,* and* IL10* gene expressions (Figures 3(a), 3(b), 3(c), 3(d), and 3(e), resp.) were similar among the MAT groups, as well as protein expressions of TNF*α*, IL17, and IL23 (Figures 3(f), 3(g), and 3(h), resp.). The anti-inflammatory cytokine IL10 was increased in MAT of CD patients when compared to respective control (*p* > 0.05; Figure 3(i)).

### 3.4. Modulation of Nuclear Transcription Factors in the Intestinal Mucosa and MAT of CD Patients

**Figure 4:**
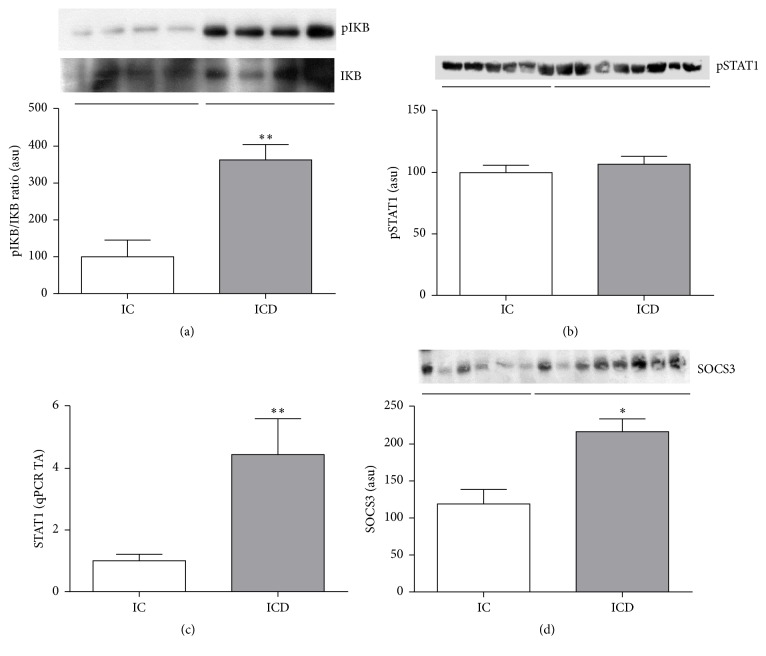
Intestinal mucosa of Crohn's disease patients shows inflammation driven by NF-KB. Higher SOCS3 protein expression counterbalances the STAT1 activation. Western blot analysis of pI*κ*B/I*κ*B ratio (a), pSTAT1 (b), and SOCS3 (d) in intestinal mucosa of CD patients (ICD Group) compared to controls (IC Group). mRNA levels (qRT-PCR) of* STAT1* (c) in intestinal mucosa of CD patients (ICD Group) compared to controls (IC Group). Each band represents one patient. For ICD, *n* = 10; for IC, *n* = 8;  ^*∗*^*p* < 0.05,  ^*∗∗*^*p* < 0.01, and ^*∗∗∗*^*p* < 0.001 versus control. ASU: arbitrary scanning unit; TA: transcription amount.

**Figure 5:**
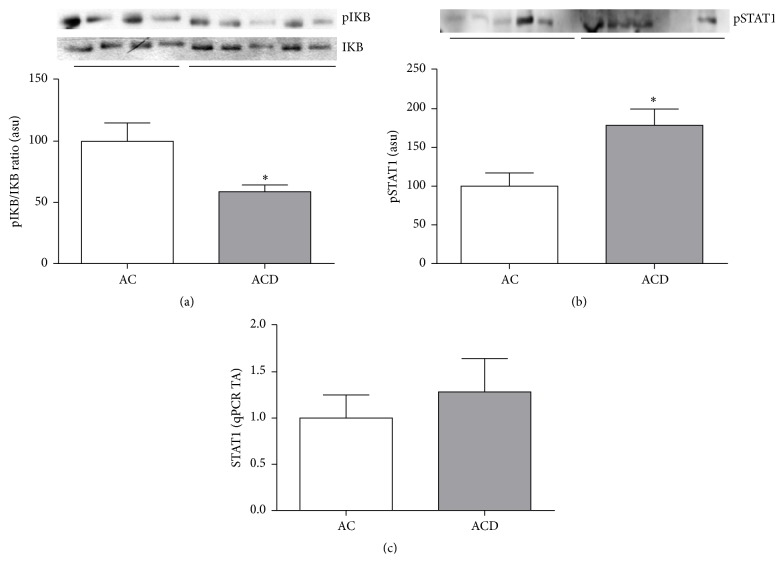
Mesenteric adipose tissue of Crohn's disease patients shows activation of STAT1 transcriptional pathway. Western blot analysis of pI*κ*B/I*κ*B ratio (a) and pSTAT1 (b) in the mesenteric adipose tissue (MAT) of CD patients (ACD Group) compared to controls (AC Group). mRNA levels (qRT-PCR) of* STAT1* (c) in MAT of CD patients (ACD Group) when compared to controls (AC Group). Each band represents one patient. For ACD, *n* = 10; and, for AC, *n* = 8,  ^*∗*^*p* < 0.05,  ^*∗∗*^*p* < 0.01, and ^*∗∗∗*^*p* < 0.001 versus control. ASU: arbitrary scanning unit; TA: transcription amount.

We evaluated the pIkB/IkB ratio by immunoblotting and it was significantly higher in the ICD Group when compared to control (*p* < 0.05; Figure 4(a)). Conversely, the pIkB/IkB ratio was lower in MAT of CD patients when compared to control group (*p* > 0.05; Figure 5(a)), showing a different pattern in the inflammatory signaling response compared to the intestinal mucosa.

Despite the increased gene expression of* STAT1* in the intestinal mucosa of CD patients compared to control group (*p* < 0.05Figure 4(c)), no differences in the phosphorylated and activated form of STAT1 (pSTAT1) were observed among the groups in intestinal mucosa (Figure 4(b)). Indeed, the SOCS3 protein, which is a downregulator of STAT pathways, was also significantly higher in ICD Group when compared to IC Group (*p* < 0.05; Figure 4(d)).

Interestingly, although there were no differences in* STAT1* gene expression, the protein content of the activated form of STAT1 (pSTAT1) was higher in ACD Group compared to AC Group (*p* < 0.05), showing that the STAT1 transcriptional pathway is activated in MAT of CD patients. Figures 5(c) and 5(b), respectively, illustrate these findings.

## 4. Discussion

The predominance of NF-kB activation in CD and STAT1 activation in UC has been reported in the literature [9, 10, 17]. Schreiber et al. [9] studied patients with active IBD and compared the expression and activation of STAT1 in endoscopic colonic biopsies. They concluded that STAT1 expression and activation are higher in UC compared to CD and controls. However, there are no studies of STAT1 activation in MAT. Schreiber et al. [10] revealed that NF-kB activation was detected in intestinal mucosa of both diseases, CD and UC, but the highest levels were seen in the* lamina propria* biopsy samples from CD patients. NF-kB is found in cytoplasm and is bound to IkB*α*, which prevents it from going into the nucleus [18]. Once this signaling pathway is activated, the protein IkB*α* is phosphorylated and degraded by the ubiquitin-proteasome pathway, releasing the NF-kB, which binds to specific DNA sequences in the nucleus, activating target genes involved in the proinflammatory response [18].

In the present study, we show that TNF*α* expression and the NF-kB signaling pathway are activated in the intestinal mucosa of CD patients when compared to controls, whereas no differences were observed in STAT1 pathway, as demonstrated by the lack of pSTAT1 modulation in ICD and IC groups. This finding can be explained by the increased levels of SOCS3 in ICD Group, which may inhibit the STAT1 activation [19] and reinforce the predominance of NF-kB pathway in CD. At least in experimental models [20], NF-kB inhibitors ameliorate colonic inflammation.

Additionally, several other proinflammatory cytokines were found to be increased in intestinal mucosa of CD. Furthermore, although higher TNF*α* protein levels were noticed in the intestinal mucosa of CD, no differences were observed in its transcriptional expression, probably due to increased stability of the gene promoter [21]. Another possibility is that high TNF*α* protein expression could induce a negative feedback of TNF*α* gene transcription. Conversely, higher STAT1 gene expression in intestinal mucosa of CD and no differences in pSTAT1 expression were observed and could be explained by a higher protein stability and protection from ubiquitination and degradation outcomes.

The increase of MAT is usually shown as a marker of an active and more aggressive CD [22–26]. MAT can have an important role in the maintenance of CD, since the altered balance between proinflammatory and anti-inflammatory factors in adipocytes could lead to the activation of the innate and adaptive immune response [27, 28]. Previous studies have demonstrated the controversy expression of important inflammatory markers in MAT of CD patients. Desreumaux and coworkers published in 1999 a study showing an increased expression of TNF*α* in the MAT of the small bowel mesentery in CD patients, suggesting the adipocytes as one of the source of TNF*α* production [29]. Our results seem controversial to the literature, once we show no differences in TNF*α* expression, along with decreased NF-kB pathway activation demonstrated by decreased pIKB/IKB ratio and increased IL10 expression in MAT of CD patients. Besides, there were no differences in IL17 and IL23 protein expressions in this tissue.

Furthermore, a study conducted by Yamamoto and collaborators [3] in 2005 showed an increase of adiponectin secretion and tissue concentration in hypertrophied MAT of CD patients. Although, in this study, the comparison was made with normal MAT from IBD patients, differently of our controls, they concluded that the MAT could serve as a barrier that prevents the spread of inflammation into the intra-abdominal space. Conversely, Rodrigues and collaborators [30] in 2012 revealed low expression of adiponectin in MAT of CD patients, which may show a deficiency in the anti-inflammatory mechanism in intestinal mesentery near the affected intestinal area during the late stages of this chronic disease, and this could help to perpetuate a state of chronic inflammation. Our results showing the increased protein levels of IL10 in MAT of CD patients corroborate with Yamamoto study; however, the controversy persists.

Additionally, our results showed no differences among protein and gene expression of TNF*α* or other proinflammatory cytokines in AC and ACD groups. Also, the pIkB/IkB ratio was lower in ACD Group compared to AC Group. No difference in STAT1 gene expression was observed in MAT; however the pSTAT1 was higher in MAT of CD patients, similar to the increase found in UC patients, as reported in the literature [31]. Although we found higher IL10 and pSTAT1 protein expression in MAT of CD, there were no differences in the transcriptional levels. This discrepancy of transcription and protein levels may be also explained by an increased stability of the gene promoter or a negative feedback of IL10 and STAT1 gene transcription induced by their respective higher protein expressions [21].

The role of MAT is still controversial in the literature as discussed above. The small diameter of MAT adipocytes of CD, as seen in severe obese patients, suggests a latent inflammatory condition in MAT [22]. Morphometric features of the adipocytes from CD MAT were already verified in the literature [4, 32]. Dias and collaborators [32] also correlated the lower diameter and area of the adipocytes with lower apoptosis, which could explain the higher number of adipocytes per field of higher magnification. Indeed, they did not find proliferation in this tissue as expected in nonobese individuals. Mature adipocytes are not able to do cell division; we can only see adipocyte proliferation in severe obesity. However, another mechanism that can lead to adipocyte number increase is by cell differentiation. This occurs through the adipogenesis pathway initiated by adipose stem cells [33, 34], which could explain the morphological and metabolic alterations in CD MAT. Recently, paracrine functions of the adipose-derived stromal vascular fraction (AD-SVF) have been studied and heterogeneous populations of undifferentiated mononuclear elements were also verified [34, 35]. Thus, in light of our results, it is still hard to confirm if MAT have an anti-inflammatory role that could attenuate the inflammation in CD patients or if a mild proinflammatory response is activated as seen in UC patients. The interaction between cytokines, adipokines, transcription factors, adipose stem cells, vascular endothelia, and adipocyte plasticity may be implicated in MAT remodeling, which certainly influences CD physiopathology.

Therefore the results of the present study reinforce the predominance of proinflammatory NF-KB pathway in the intestinal mucosa of CD patients. Additionally, for the first time, we showed that the inflammatory status of MAT in CD is mediated by STAT1 activation. On the other hand, this activation may lead to increase of anti-inflammatory cytokines, such as IL10. According to this study, MAT may play an important role in the pathophysiology and/or activity of CD. Indeed, the knowledge of the molecular signaling activated, along with the transcription factors and cytokine expression in different tissues, such as MAT and intestinal mucosa, is important to understand the physiopathology of CD and also to search for new drugs that address tissue-specific differences.

## Supplementary Material

Total protein staining confirmed equal loading in Western blot analysis of TNFα, IL1β, IL10, IL17, IL23, pSTAT1, SOCS3 expressions in intestinal mucosa (A, B) and in mesenteric adipose tissue (C, D).
