# Supplementary material for: Transcriptional and Molecular Pathways Activated in Mesenteric Adipose Tissue and Intestinal Mucosa of Crohn's Disease Patients
Source: Int J Inflam. 2017 Apr 9;2017:7646859. doi: 10.1155/2017/7646859 (PMC5401739; doi:10.1155/2017/7646859)

Figure S1. Ponceau-S staining of the Western blot membranes used as loading controls. Total protein staining confirmed equal loading in Western blot analysis of TNFα, IL1β, IL10, IL17, IL23, pSTAT1, SOCS3 expressions in intestinal mucosa (A, B) and in mesenteric adipose tissue (C, D) as presented in Figures 3, 5, 6 and 7.

Supplementary Figure

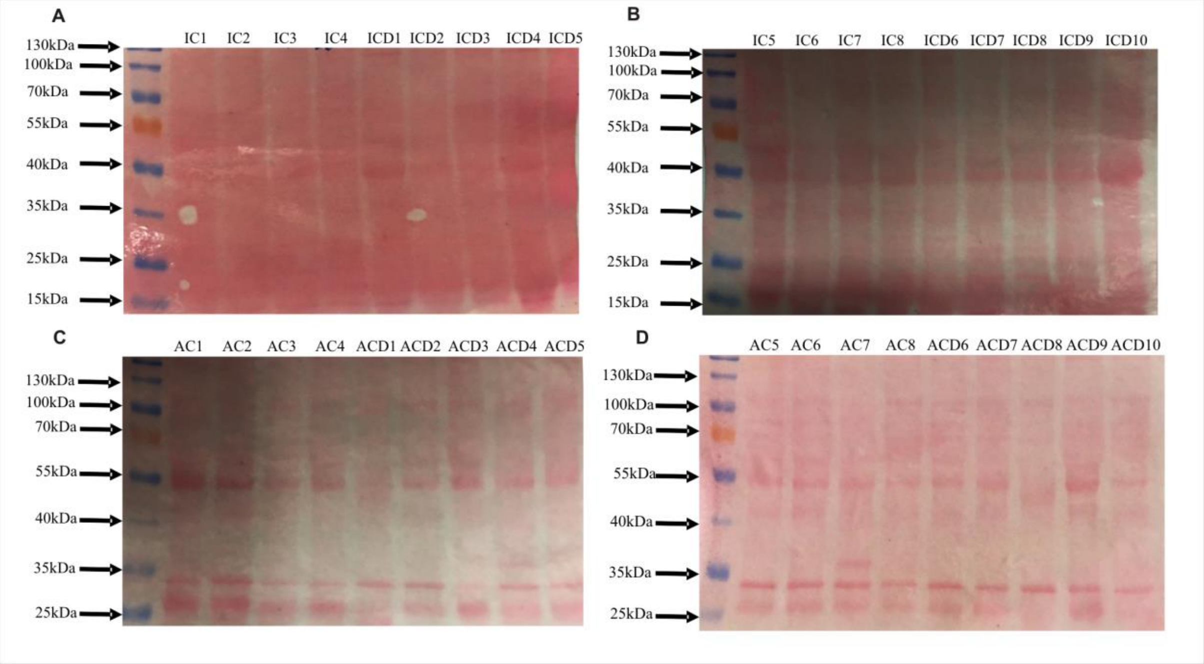

Supplement: Supplementary file 1 — Total protein staining confirmed equal loading in Western blot analysis of TNFα, IL1β, IL10, IL17, IL23, pSTAT1, SOCS3 expressions in intestinal mucosa (A, B) and in mesenteric adipose tissue (C, D). [file 7646859.f1.pdf]
